# Telomere length variation in tumor cells and cancer‐associated fibroblasts: potential biomarker for hepatocellular carcinoma

**DOI:** 10.1002/path.4961

**Published:** 2017-10-13

**Authors:** Li‐Jie Ma, Xiao‐Ying Wang, Meng Duan, Long‐Zi Liu, Jie‐Yi Shi, Liang‐Qing Dong, Liu‐Xiao Yang, Zhi‐Chao Wang, Zhen‐Bin Ding, Ai‐Wu Ke, Ya Cao, Xiao‐Ming Zhang, Jian Zhou, Jia Fan, Qiang Gao

**Affiliations:** ^1^ Department of Liver Surgery and Transplantation, Liver Cancer Institute, Zhongshan Hospital, and Key Laboratory of Carcinogenesis and Cancer Invasion (Ministry of Education) Fudan University Shanghai PR China; ^2^ Cancer Research Institute, Xiangya School of Medicine Central South University Hunan PR China; ^3^ Key Laboratory of Molecular Virology and Immunology, Institute Pasteur of Shanghai Chinese Academy of Sciences Shanghai PR China; ^4^ State Key Laboratory of Genetic Engineering Fudan University Shanghai PR China

**Keywords:** hepatocellular carcinoma, telomere length, TERTp, FISH, prognosis

## Abstract

The role of telomere dysfunction and aberrant telomerase activities in hepatocellular carcinoma (HCC) has been overlooked for many years. This study aimed to delineate the variation and prognostic value of telomere length in HCC. Telomere‐specific fluorescence in situ hybridization (FISH) and qPCR were used to evaluate telomere length in HCC cell lines, tumor tissues, and isolated non‐tumor cells within the tumor. Significant telomere attrition was found in tumor cells and cancer‐associated fibroblasts (CAFs) compared to their normal counterparts, but not in intratumor leukocytes or bile duct epithelial cells. Clinical relevance and prognostic value of telomere length were investigated on tissue microarrays of 257 surgically treated HCC patients. Reduced intensity of telomere signals in tumor cells or CAFs correlated with larger tumor size and the presence of vascular invasion (p < 0.05). Shortened telomeres in tumor cells or CAFs associated with reduced survival and increased recurrence, and were identified as independent prognosticators for HCC patients (p < 0.05). These findings were validated in an independent HCC cohort of 371 HCC patients from The Cancer Genome Atlas (TCGA) database, confirming telomere attrition and its prognostic value in HCC. We also showed that telomerase reverse transcriptase promoter (TERTp) mutation correlated with telomere shortening in HCC. Telomere variation in tumor cells and non‐tumor cells within the tumor microenvironment of HCC was a valuable prognostic biomarker for this fatal malignancy. © 2017 The Authors. *The Journal of Pathology* published by John Wiley & Sons Ltd on behalf of Pathological Society of Great Britain and Ireland.

## Introduction

Hepatocellular carcinoma (HCC) is the third most common cause of cancer deaths and the fourth most common cancer worldwide 1. Chronic liver damage, such as that caused by chronic hepatitis, liver cirrhosis, and fatty liver disease, is closely associated with the occurrence of HCC 2. Recent advances in sequencing technologies have enabled the identification of multiple driver genetic alterations and pathways implicated in hepatocarcinogenesis and tumor progression 3. This may help us to develop new targets and biomarkers that ultimately improve clinical decision‐making and patient outcomes. Of note, among the recurrent oncogenic mutations identified in HCC, the most prevalent is telomerase reverse transcriptase (*TERT*) promoter (TERTp) mutations, which occurs in up to 60% of patients, highlighting the importance of telomere biology in HCC molecular pathogenesis 4.

Telomeres are specialized structures located at the ends of chromosomes, playing a critical role in maintaining chromosomal integrity and stability. In normal cells, continuous telomere shortening with each cell division triggers DNA damage responses and initiates irreversible cellular senescence or apoptosis 5. Alternatively, chromosome instability and DNA damage‐induced genetic mutations due to shortened telomeres may result in neoplastic transformation 6, 7. It has been proposed that permanent proliferation of cancer cells depends on the maintenance of telomere length 8. To counteract telomere shortening, up to 90% of human cancers, including HCC, reactivate telomerase 9. Data have accumulated that tumor telomeres are shorter than normal tissues in the majority of human cancers 10, 11, 12, while longer telomeres in sarcomas and gliomas were also observed 13. Likewise, there are conflicting results regarding the impact of telomere length on cancer susceptibility and survival. For example, short telomeres predicted a poor prognosis in chronic lymphocytic leukemia and colorectal cancer, but a reduced death risk in esophageal cancer 14. Intrinsic biological features in each cancer demand that the clinical significance of telomere length needs cancer‐specific investigation. Although HCC has the second highest frequency of *TERT* promoter mutations among 31 cancer types 13, the clinical relevance of telomere attrition or elongation in HCC remains unknown.

The unique signature of the liver microenvironment, characterized by a chronic inflammatory state and dysregulated immune response, was associated with the biological behavior of HCC 15. Within the HCC microenvironment, cancer‐associated fibroblasts (CAFs) and tumor‐infiltrating lymphocytes (TILs) are of paramount importance. Experimental and clinical evidence demonstrated that interactions between CAFs or TILs and tumor cells could promote HCC progression and metastasis through various mechanisms 16, 17. A recent study in prostate cancer suggested that investigation of telomere length in cancer‐associated stromal cells is feasible and is significant for predicting cancer behavior 18. Large‐scale prospective studies suggested that telomere attrition in peripheral blood leukocytes correlated not only to poor prognosis of a group of human cancers 19, 20 but also with increased mortality in the general population 21. Thus, it is rational to speculate that telomere attrition or elongation in CAFs or TILs would harbor significant clinical value in HCC.

Based on the above information, we evaluated telomere lengths in tumor cells, CAFs, and TILs in a large cohort of HCCs using telomere‐specific fluorescence *in situ* hybridization (FISH) and qPCR. The recently developed FISH‐based method enables accurate measurement of telomere length at single‐cell resolution, greatly facilitating such analysis 18. We found that shortened telomeres in tumor cells and CAFs, rather than in TILs, were independently and significantly associated with the clinical outcome of HCC patients.

## Materials and methods

### Patients and sample collection

A cohort of 257 HCC patients who received curative hepatectomy at Zhongshan Hospital (Fudan University, China) from January to December 2007 was enrolled. This study was conducted after obtaining informed consent forms from patients and ethical approval from Zhongshan Hospital Research Ethics Committee. The inclusion and exclusion criteria for patients, therapeutic modalities, and postoperative surveillance according to a uniform guideline have been described previously 22. The clinicopathologic features are provided in the supplementary material, Table S1, and patient follow‐up is included in the Supplementary materials and methods. Tissue microarrays were constructed as described previously 23. Details are included in the supplementary material, Supplementary materials and methods.

### Cell lines

Five human HCC cell lines (Huh7, SMMC‐7721, MHCC‐97 L, MHCC‐97H, and MHCC‐LM3), an immortalized human liver cell line (L‐02), and the cervical cancer cell line Hela were used as described in the supplementary material, Supplementary materials and methods.

### Cell isolation, purification, and phenotypic characterization

Isolation of CD45^+^ leukocytes and α‐SMA^+^ fibroblasts from peritumoral liver and HCC tissues using MicroBeads and an MS Column (Miltenyi Biotec, Bergisch Gladbach, Germany) was performed according to the manufacturer's instructions. Phenotypic characterization of the isolated cells was conducted using flow cytometry as previously described 24. Details are included in the supplementary material, Supplementary materials and methods.

### Measurement of relative telomere length by real‐time quantitative PCR (qPCR) and quantitative fluorescent in situ hybridization (FISH)

Genomic DNA was extracted from paired peritumoral liver and HCC tissues from 24 patients, as well as isolated leukocytes and fibroblasts from ten patients. mRNA was extracted from paired peritumoral liver and HCC tissues from another 64 patients. Relative telomere length and mRNA expression were measured by real‐time qPCR as previously described 25.

The assessment of telomere length was conducted by telomere FISH for telomeric DNA as previously described 18, 26. Sections were imaged by an Olympus BX51 fluorescence microscope equipped with a UIS2 optical system and Kohler illuminator (OLYMPUS, Tokyo, Japan) using a 40X/1.42 NA UPLFLN lens with correction collar. Quantification of the digitized fluorescent telomere captures was performed applying the open source JAVA‐based image analysis software package ImageJ as previously described 15. Details are reported in the supplementary material, Supplementary materials and methods.

### Statistical analyses

Data are expressed as the mean ± SEM, and error bars in the figures refer to SEM, median, and interquartile range (IQR). The analysis of association between variables was conducted using the Mann–Whitney *U*‐test, Student *t*‐test, chi‐square test, or one‐way ANOVA test when appropriate. Univariate analysis was based on the Kaplan–Meier method using the log‐rank test. Cox proportional hazards regression was used for multivariate analyses. SPSS software (IBM, Armonk, NY, USA) and Graph Pad Prism 6.0 (Graph Pad Software Inc, San Diego, CA, USA) were applied for data analyses. Statistical tests were two‐sided, with *p* < 0.05 considered significant.

## Results

### Assessment of telomere length in HCC cell lines

**Figure 1:**
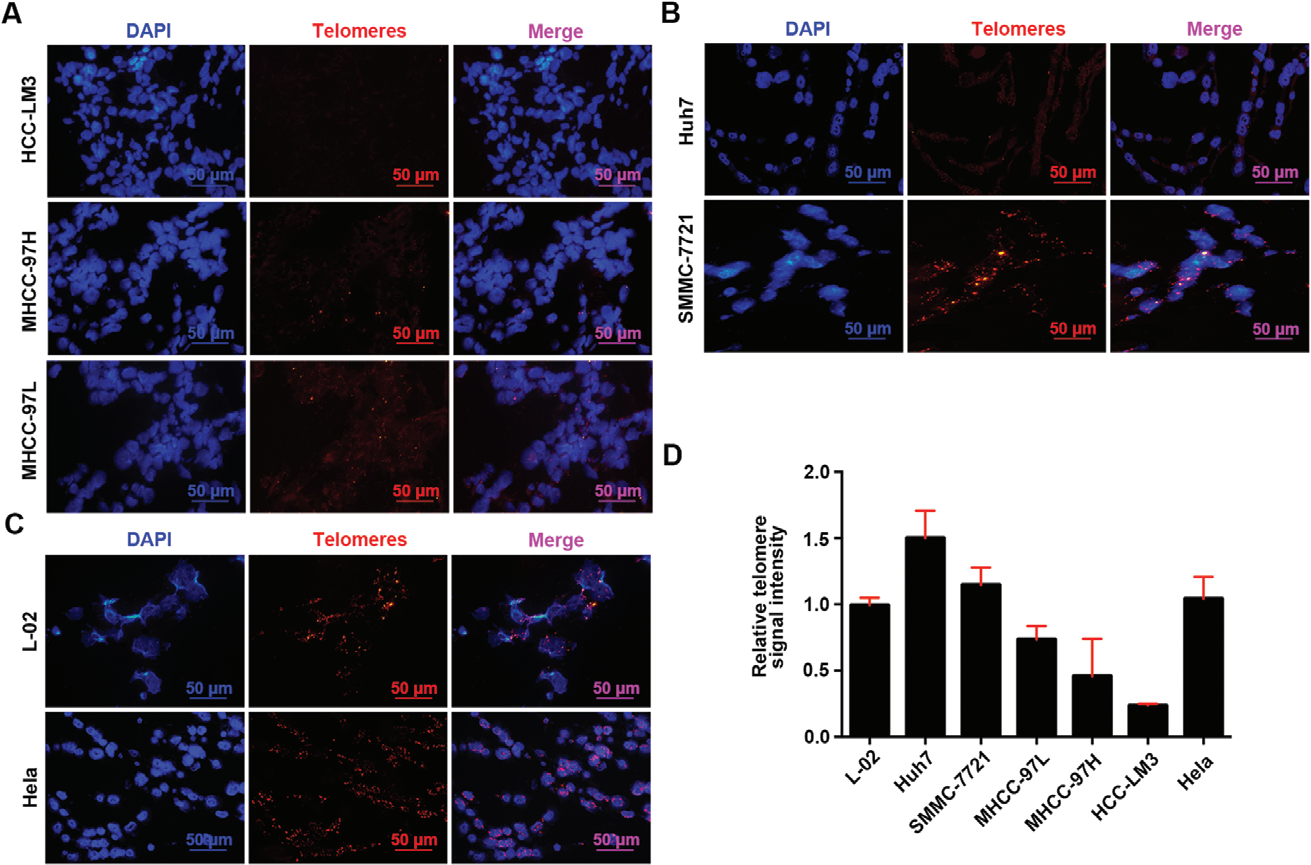
Telomere‐specific FISH in HCC cell lines. Representative FISH images of (A) MHCC‐97 L, MHCC‐97H, and HCC‐LM3; (B) Huh7 and SMMC‐7721; and (C) L‐02 and Hela cells. Left panels: 4',6‐diamidino‐2‐phenylindole (DAPI) (blue) was used to identify nuclei. Middle panels: Cy3‐PNA telomere‐probe fluorescence. Right panels: merged images (original magnification ×40). (D) Statistics of FISH telomere signal intensity.

To test the specificity and validity of the Cy3‐labeled PNA probe, and to develop a series of positive controls for telomere length assessment in paraffin‐embedded tissue sections, we first evaluated telomere length in seven cell lines. Each cell line was fixed in formalin and embedded in paraffin to imitate standard slides of patients' tumor tissue. Representative images of the hybridization reaction localizing the fluorescent telomeric PNA probe in each cell line are shown in Figure 1A–C. Procedures for measuring telomere length by ImageJ are shown in the supplementary material, Figure S1A, B, as previously described 18. The highly metastatic cell lines (HCC‐LM3, MHCC‐97H, and MHCC‐97 L) (Figure 1A) showed minimal or no intensities of telomere FISH, while cells with low metastatic capability (Huh7 and SMMC‐7721) (Figure 1B) and the normal liver cell line L‐02 (Figure 1C) showed strong intensities, indicating the involvement of telomere length in HCC aggressiveness. In addition, Hela cells showed a high telomere signal (Figure 1C). Quantitative analysis of telomere length in cell lines is summarized in Figure 1D.

### Telomere FISH enabled high‐resolution assessment of telomere length in HCC tissue at single‐cell resolution

**Figure 2:**
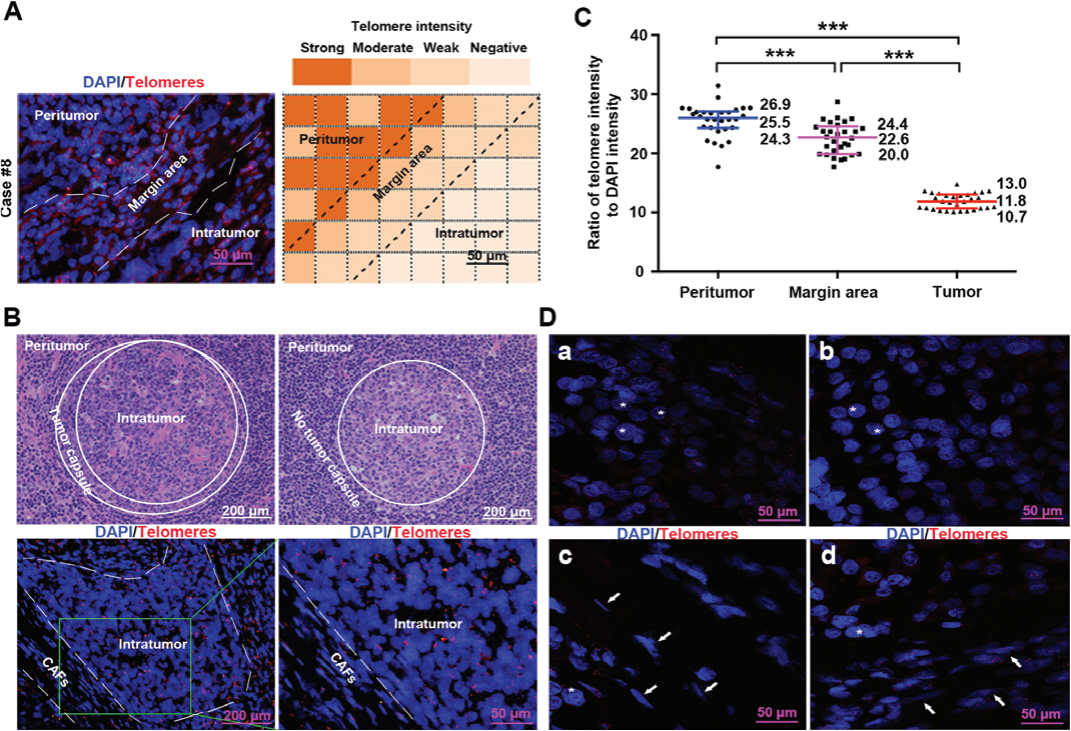
Telomere‐specific FISH in HCC tissues. (A) Left: representative image of telomere‐specific FISH on whole slides to elucidate the sub‐location and micro‐anatomic distribution of telomere signals (original magnification ×40). Right: the same section is divided into 48 grids, colored according to the relative telomere density. (B) Top: representative H&E staining of HCC with (left) or without (right) a tumor capsule (original magnification ×20). Bottom: FISH staining in the case with a tumor capsule. White dotted lines highlight the margin area (original magnification ×40). (C) Telomere‐FISH intensity quantification (n = 30). Lines indicate the 25th, 50th, and 75th percentiles. ***p < 0.001. (D) Representative examples of telomere length variation in tumor cells and CAFs in TMA. (a) Strong telomere signals in cancer cells; (b) weak telomere signals in cancer cells; (c) extremely short telomeres in CAFs; (d) long telomeres in CAFs. Asterisks indicate cancer cells and arrows indicate CAFs (original magnification ×40).

Quantification of specific telomere‐FISH signals in cells and tissues is linearly proportional to telomere length, and differences of telomere length among cells and tissues can be evaluated via quantitative image analysis 18. First, telomere‐specific FISH was applied on whole slides from 30 HCC samples to elucidate the sub‐location and microanatomic distribution of telomere signals in peritumor, tumor margin, and intratumor areas (Figure 2A, B). Telomere signals were higher in peritumor areas (median 25.5; IQR 24.3–26.9) than in intratumor areas (median 11.8; IQR 10.7–13.0) and tumor margins (median 22.6; IQR 20.0–24.4) (Figure 2C).

**Figure 3:**
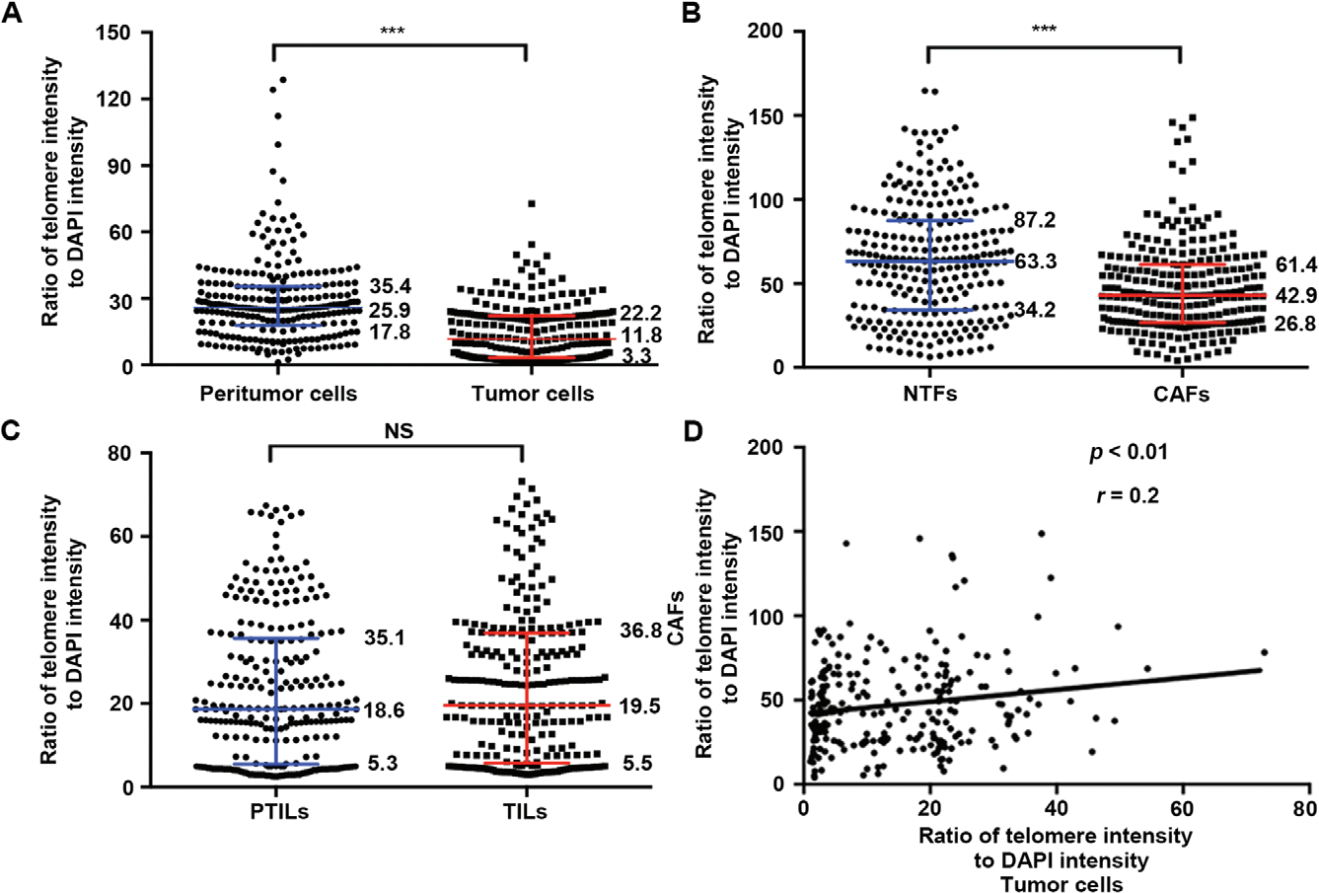
Quantitative results of telomere‐specific FISH in 257 HCCs. (A) Telomere signals in peritumor liver cells and HCC tumor cells (n = 257). (B) Telomere signals in NTFs and CAFs (n = 257). (C) Telomere signals in PTILs and TILs (n = 257). (A–C) Lines indicate the 25th, 50th, and 75th percentiles, with their respective values provided. ***p < 0.001; NS, p = 0.587. (D) The telomere signal of tumor cells correlated with that of CAFs (n = 257; r = 0.2, p < 0.01).

FISH was then performed on TMAs containing 257 HCC samples to elucidate telomere lengths in paired peritumor and intratumor areas. We calculated eight cell types separately along with H&E staining for each patient, i.e. tumor cells, peritumor liver cells, CAFs, non‐tumoral fibroblasts (NTFs), TILs, peritumor‐infiltrating lymphocytes (PTILs), peritumor bile duct epithelial cells (P‐BDECs), and tumor bile duct epithelial cells (T‐BDECs) (supplementary material, Figure S1C, D). Figure 2D shows representative images of telomere signals in tumor cells and CAFs at single‐cell resolution. Consistent with telomere shortening in other solid tumors 10, 11, 25, telomere signals were less intense (i.e. shorter telomere length) in tumor cells than in adjacent liver cells (*p* < 0.001; Figure 3A). In total, 164 out of 257 samples (63.81%) displayed significantly less intense telomere signals among tumor cells compared with corresponding normal liver cells (supplementary material, Figure S2A).

Likewise, 157 of 257 (61.08%) exhibited telomere signal diminution in CAFs compared with paired NTFs (*p* < 0.001; Figure 3B and supplementary material, Figure S2B). Diffuse or aggregated infiltration of lymphocytes was found in peritumor areas and tumor margins, with less abundant lymphocytes in intratumor areas (supplementary material, Figure S2C). Nevertheless, no obvious differences were found in telomere lengths in TILs and PTILs (*p* = 0.587; Figure 3C). Intrahepatic bile ducts were detected in intratumor areas of only 92 cases, but in the peritumor area of 168 cases (supplementary material, Figure S2D). Differences in telomere length were hard to find in BDECs between paired intratumor and peritumor areas (data not shown). The telomere length in tumor cells, CAFs, and TILs had a wide dynamic IQR.

We found that telomere length tends to be synchronously decreased in tumor cells and CAFs. A positive correlation (*p* < 0.01, *r* = 0.2) was revealed between the intensities of telomeres in the two cell types (Figure 3D), which implied potential cooperation between them in a cancer‐promoting effect. To confirm the result, the relative telomere length (RTL) of tumor and peritumor tissue pairs (*n* = 24) as well as isolated CAF and NTF pairs (*n* = 10) was assessed by qPCR, which indicated consistent results with FISH (supplementary material, Table S2). In brief, the RTL of peritumor tissues (median 0.92; IQR 0.89–1.02) was higher than that of tumor tissues (median 0.45; IQR 0.35–0.55; *p* < 0.001) (supplementary material, Figure S3A). Likewise, the RTL decreased in CAFs (median 1.50; IQR 1.15–1.64) compared with NTFs (median 2.06; IQR 1.68–2.34; *p* < 0.01) (supplementary material, Figure S3B). In addition, the RTL of leukocytes isolated from ten patient samples showed no differences between PTILs (median 1.66; IQR 1.05‐1–81) and TILs (median 1.64; IQR 1.02–1.97; *p* = 0.945) (supplementary material, Figure S3C). The statistics of telomere‐specific FISH variables are shown in the supplementary material, Table S2. Since telomere lengths are maintained by reactivation of telomerase in the majority of human malignancies 27, we also detected the mRNA level of telomerase by real‐time RT‐PCR in 64 HCC patients and found a positive correlation between *TERT* mRNA level and RTL (*p* < 0.0001, *r* = 0.806) (supplementary material, Figure S3D).

### Shorter telomeres in HCC cells or CAFs correlate with poor prognosis

**Figure 4:**
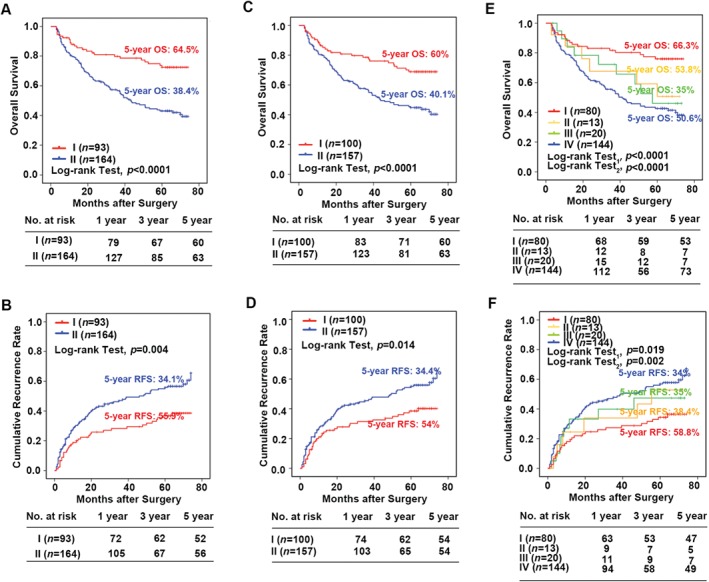
Telomere length is associated with survival and recurrence of HCC. (A–F) Kaplan–Meier curves according to telomere signal intensity. (A, B) Tumor cells; (C, D) CAFs; (E, F) the combination of telomere signals in tumor cells and CAFs. P values are based on the log‐rank test. Log‐rank test_1_: comparing survival and recurrence across all four groups; log‐rank test_2_: comparing survival and recurrence between patients with shorter/shorter combination and patients with longer/longer combination of telomere length (see text for groupings). RFS, recurrence‐free survival.

We then focused on whether the telomere length had prognostic value and clinical relevance. We first defined samples with lower telomere signals in tumor cells than in paired peritumor liver cells as the shorter group, and higher telomere signals as the longer group (supplementary material, Figure S3E). Likewise, patients were grouped into shorter and longer according to the telomere signals of CAFs and NTFs (supplementary material, Figure S3F). Kaplan–Meier analysis demonstrated that the median OS time was 44.1 and 59.8 months for patients with shorter and longer telomeres in tumor cells, respectively (*p* < 0.0001, Figure 4A). The median TTR was 41.7 and 53.8 months for patients with shorter and longer telomeres in tumor cells, respectively (*p* = 0.004, Figure 4B). Similarly, patients with shorter telomeres in CAFs had a poorer prognosis for both OS (44.7 versus 58.3 months, *p* < 0.001, Figure 4C) and TTR (42.5 versus 52.1 months, *p* = 0.014, Figure 4D; log‐rank test for each comparison).

Alternatively, the influence of telomere length was evaluated within intratumor and peritumor areas. Using the median telomere length as the cut‐off (tumor cell median = 11.8, IQR 3.3–22.2; CAF median = 42.9, IQR 26.8–61.4), patients were also classified into shorter and longer groups. Kaplan–Meier analysis demonstrated that the median OS time was 43.2 and 56.4 months for patients with shorter and longer telomeres in tumor cells, respectively (*p* < 0.0001, supplementary material, Figure S4A). The median TTR was 40.8 and 51.3 months for patients with shorter and longer telomeres in tumor cells, respectively (*p* = 0.007, supplementary material, Figure S4B). Likewise, we also found that patients with shorter telomeres in CAFs had a significantly poorer OS (44.7 versus 58.3 months, *p =* 0.008, supplementary material, Figure S4C) and TTR (42.2 versus 50.4 months, *p* = 0.048, supplementary material, Figure S4D; log‐rank test for each comparison). However, telomere variation in peritumoral liver cells and NTFs showed no prognostic significance.

Furthermore, we evaluated the relationship between patient clinicopathologic features and telomere length in tumor cells or CAFs. Reduced intensity of telomere signals in tumor cells correlated positively with larger tumor size (*p <* 0.001), the presence of vascular invasion (*p =* 0.046), poor tumor differentiation (*p* = 0.041), and advanced tumor stages (*p* = 0.001). Similarly, shorter telomeres in CAFs were associated with larger tumor size (*p =* 0.032) and the presence of vascular invasion (*p* = 0.031) (Table 1). However, no significance was found between telomere variation and other features, such as hepatitis virus, gender, cirrhosis, tumor encapsulation, and tumor number.

**Table 1:** Correlation of clinicopathologic features with telomere length in tumor cells and CAFs in HCC patients (n = 257)

	Telomeres in tumor cells			Telomeres in CAFs		
Characteristic	Longer	Shorter	*p* *	Longer	Shorter	*p* *
Age (years)						
≤ 51	61	74	0.679	48	87	0.446
> 51	52	70		49	73	
Gender						
Female	19	19	0.417	18	20	0.185
Male	94	125		79	140	
HBsAg						
Negative	5	4	0.710†	5	4	0.440†
Positive	108	140		92	156	
HCVAb						
Negative	110	138	0.754†	95	153	0.530†
Positive	3	6		2	7	
AFP (ng/ml)						
≤ 20	51	44	**0.016**	30	65	0.118
> 20	62	100		67	95	
ALT (U/l)						
≤ 75	101	129	0.958	86	144	0.734
> 75	12	15		11	16	
γ‐GT (U/l)						
≤54	27	27	0.315	26	28	0.076
>54	86	117		71	132	
Liver cirrhosis						
No	14	11	0.202	13	12	0.122
Yes	99	133		84	148	
Tumor size (cm)						
≤ 5	77	59	**0.001**	43	93	**0.032**
> 5	36	85		54	67	
Tumor number						
Single	91	121	0.464	78	134	0.495
Multiple	22	23		19	26	
Vascular invasion						
No	81	86	**0.046**	71	96	**0.032**
Yes	32	58		26	64	
Tumor encapsulation						
None	45	69	0.195	41	73	0.599
Complete	68	75		56	87	
Tumor differentiation						
I + II	87	94	**0.041**	70	111	0.634
III + IV	26	50		27	49	
TNM stage						
I	50	95	**0.001**	48	97	0.081
II + III	63	49		49	63	

Numbers in bold indicate that the P value is significant.

* Pearson chi‐square test.

† Chi‐square with Yates' correction.

### Combined influence of telomere length in tumor cells and CAFs on the risk of HCC death and recurrence

Next, we classified the 257 HCC cases into four groups based on telomere lengths in tumor cells and CAFs. Group I contained cases with longer telomeres in both tumor cells and CAFs; group II included cases with longer telomeres in tumor cells but shorter telomeres in CAFs; group III included cases with shorter telomeres in tumor cells but longer telomeres in CAFs; group IV contained cases with shorter telomeres in both tumor cells and CAFs. Applying Kaplan–Meier analysis, patients in group IV had the shortest OS (median 43.5 months) and TTR (median 41.3 months), whereas patients in group I had the longest OS (median 61.2 months) and TTR (median 54.7 months) (Figure 4E, F). For OS, comparing the four combinations, *p* < 0.0001; comparing the shorter/shorter combination with the longer/longer combination, *p* < 0.0001; for TTR, comparing the four combinations, *p* = 0.019; and comparing the shorter/shorter combination with the longer/longer combination, *p* = 0.002.

### Multivariate analyses

To illustrate whether the prognostic significance of telomere length was independent of clinical variables, clinicopathologic features showing significance by univariate analysis were adopted as covariates when performing multivariate Cox proportional hazard analyses (Table 2 and supplementary material, Tables S3 and S4). Shortened telomeres in HCC cells were an independent prognostic factor for both reduced overall survival (OS) and time to recurrence (TTR). Patients with shorter telomeres harbored a 2.555‐fold higher risk of death (HR 2.555; 95% CI 1.616–4.039, *p* < 0.001) and were more likely to suffer from relapse (HR 1.755; 95% CI 1.168–2.637, *p* = 0.007) than patients with longer telomeres. For telomere length in CAFs, multivariate analysis also revealed significant differences in OS and TTR between the two groups. In addition, the combination of shorter telomere in tumor cells and CAFs was an independent prognostic factor for both OS and TTR by multivariate analysis.

**Table 2:** Univariate and multivariate analyses of association with overall survival (OS) and time to recurrence (TTR) of telomere length in tumor cells and CAFs (n = 257)

	OS				TTR			
	Univariate	Multivariate			Univariate	Multivariate		
Variable	*p*	HR	95% CI	*p*	*p*	HR	95% CI	*p*
Tumor cells (shorter versus longer)	**< 0.001**	**2.555**	**1.616–4.039**	**< 0.001**	**0.002**	**1.755**	**1.168–2.637**	**0.007**
CAFs (shorter versus longer)	**< 0.001**	**2.219**	**1.444–3.411**	**< 0.001**	**0.010**	**1.610**	**1.086–2.388**	**0.018**
Combination of tumor cells and CAFs*								
Overall	**< 0.001**	NA	NA	**< 0.001**	**0.024**	NA	NA	**0.022**
II versus I	0.088	1.268	0.496–3.237	0.620	0.317	0.990	0.400–2.447	0.982
III versus I	**0.041**	1.047	0.460–2.380	0.913	0.359	0.764	0.338–1.731	0.519
IV versus I	**< 0.001**	**2.633**	**1.566–4.427**	**< 0.001**	**0.002**	**1.716**	**1.096–2.686**	**0.018**

Numbers in bold indicate that the P value is significant.

* Patients were divided into four groups based on their telomere densities of tumor cells and CAFs: group I, longer telomeres in tumor cells and longer telomeres in CAFs; group II, longer telomeres in tumor cells and shorter telomeres in CAFs; group III, shorter telomeres in tumor cells and longer telomeres in CAFs; group IV, shorter telomeres in tumor cells and shorter telomeres in CAFs. For details, see the supplementary material, Tables S3 and S4.

NA = not applicable.

### Independent validation using TCGA data

**Figure 5:**
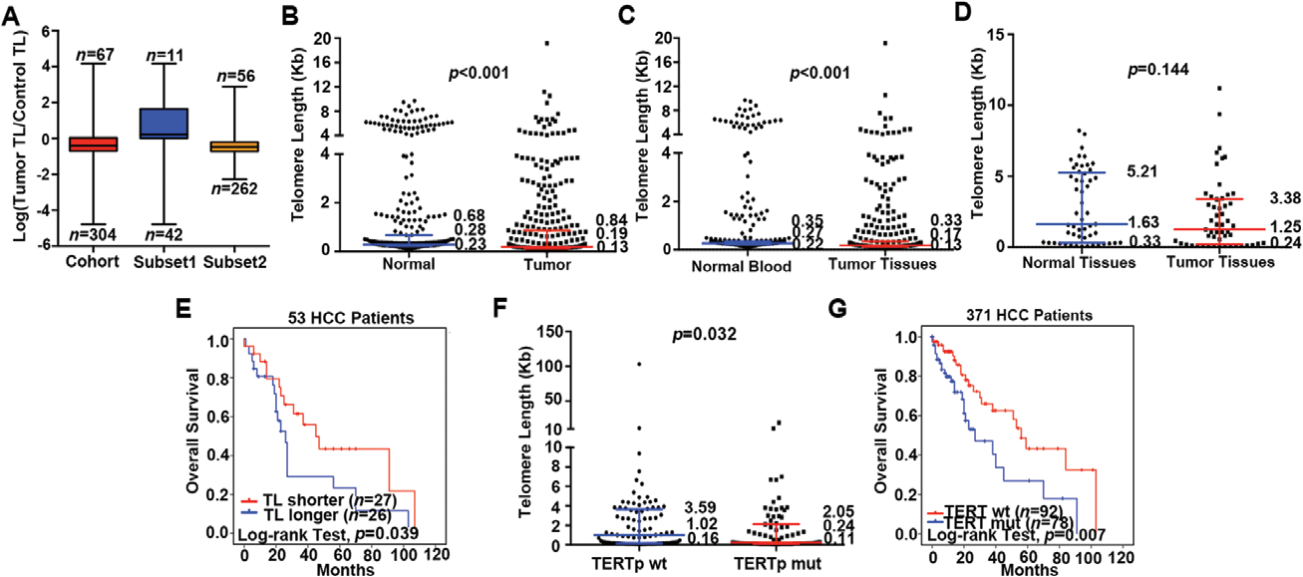
Telomere length in the TCGA cohort. (A) Telomere lengths in the entire cohort, n = 371, in subset 1 (matched tumor tissues and normal liver tissues, n = 53) and in subset 2 (matched tumor tissues and normal blood samples, n = 318). Numbers at the top and bottom show HCC cases with longer and shorter telomere lengths than paired normal, respectively. (B) Telomere length in matched tumor tissues and normal controls (n = 371). (C) Telomere length in matched tumor tissues and normal blood samples (n = 318). (D) Telomere length in matched tumor tissues and normal liver tissues (n = 53). (E) Kaplan–Meier curves of OS according to telomere length in the subset of 53 patients with tumor tissue/normal liver tissue. (F) Distribution of tumor telomere length according to the presence and absence of TERTp mutations (n = 371). (G) Kaplan–Meier curves of OS according to TERTp mutation status log‐rank test (comparing TERTp mut and TERTp wt). (A–D, F) Error bars indicate interquartile range. NT, normal tissue; TT, tumor tissue; NB, normal blood; mut, mutation; wt, wild type.

To validate the clinical significance of telomere length, we analyzed the variation and the prognostic value of telomere length in an independent cohort of 371 HCC patients from TCGA 13. This cohort included 318 HCC patients with paired tumor and blood samples, as well as 53 HCC paired tumor and normal liver tissues (Figure 5A). Telomere length was quantified based on the linear mixed modeling adjusted high‐confidence whole‐genome sequencing (*n* = 54) and whole‐exome sequencing (*n* = 317) data. Consistent with our results, 81.94% of 371 samples displayed telomere attrition in tumor tissues (median 0.19; IQR 0.13–0.84) compared with normal controls (median 0.28; IQR 0.23–0.68) (*p* < 0.001; Figure 5B). Of note, 262 of 318 paired tumor and blood samples (82.38%) showed significant telomere attrition in tumor tissues (median 0.17; IQR 0.13–0.33) compared with the blood samples (median 0.27; IQR 0.22–0.35) (*p* < 0.001; Figure 5C). Likewise, 79.25% of 53 HCC patients with paired tumor and normal liver tissues had shorter telomeres in tumor (median 1.25; IQR 0.23–3.38) than in paired normal tissues (median 1.63; IQR 0.32–5.21), although no statistically significant difference was detected (*p* = 0.144; Figure 5D), probably due to the small sample size.

Using the median of telomere length in tumor tissues as the cut‐off value in the 53 cases with paired tumor and normal liver tissues, a significant difference in OS was revealed between patients with shorter and longer telomeres (median 20 versus 31 months, *p* = 0.039, log‐rank test; Figure 5E). However, no significant difference was seen in the 318 cases with paired tumor and blood samples (median 11 versus 11 months, *p =* 0.823). This subset of HCC patients received various treatments, including resection (*n* = 259), radiotherapy (*n* = 9), and ablation and embolization (*n* = 31) (19 patients' treatment data were unavailable) 10. Thus, it is possible that variations in treatment selection contribute to the lack of statistical significance for prognosis among the 318 patients.

*TERT* promoter (TERTp) mutations are associated not only with increased transcription of the catalytic subunit but also with up‐regulated telomerase activity in tumor tissues 13. We evaluated the relationship between TERTp mutation and telomere length in the 371 HCC patients. TERTp mutation correlated positively and significantly with telomere shortening (*p =* 0.032; Figure 5F). In addition, patients with TERTp mutations showed a shorter OS compared with patients without TERTp mutations (*p* = 0.007, log‐rank test; Figure 5G). Altogether, the prognostic value of shortened telomeres was validated in the independent cohort of 371 HCC patients.

## Discussion

It is well recognized that telomere dysfunction plays a critical role in cancer initiation and progression, although the exact underlying mechanisms still need in‐depth investigation. Herein, we provided a catalogue of telomere variation in tumor cells and non‐tumor cells within the tumor microenvironment of HCC by telomere‐specific FISH and qPCR. Telomere attrition was found in tumor cells and CAFs, but not in TILs or BDECs. Of note, our data revealed that shortened telomeres in tumor cells or CAFs were independently and significantly associated with poorer postoperative outcome in HCC patients. The results were validated in an independent cohort of 371 HCCs from the TCGA database.

Telomere shortening has been reported in various human cancers using FISH 28, 29, 30, 31. Shortened telomeres were also found in peripheral blood leukocytes (PBLs) in some human cancers using qPCR 32, 33. Similarly, several studies have demonstrated that telomeres are shorter in HCC compared with peritumor tissues measured by Southern blot or qPCR 34, 35, 36. Investigators had also revealed that telomere shortening occurs in chronic liver diseases 35. However, contrasting conclusions also exist. Higher RTL was found to be associated with aggressive tumor behavior and higher grade in HCC mainly using qPCR 37, 38. RTL of PBLs in HCC was found to be the longest, followed by chronic hepatitis B (CHB) and healthy controls as the shortest, as measured by qPCR 37. The discrepancy in these results may result from the differences in detection methods, specimen sources, and therapeutic schedules. In this study, both qPCR and FISH assays confirmed that telomere shortening occurred in tumor cells and CAFs in over 60% of HCCs compared with their normal counterparts, independently validated in the TCGA dataset. More recently, WES and WGS data showed shorter telomeres in tumors than in normal tissues among 29 of 31 cancer types 13. Thus, telomere shortening should be considered as a common phenomenon in human cancers including HCC. In this respect, there is accumulating data that telomere shortening correlates with cancer susceptibility, such as breast cancer, head and neck squamous cell cancer, and gastrointestinal tumors 39, 40, 41.

With regard to the prognostic value of telomere length, previous data were conflicting. For example, short telomeres correlated with poor prognosis in chronic lymphocytic leukemia, colorectal cancer, and prostate cancer 18, but with favorable prognosis in esophageal and breast cancers 42, 43. For HCC, two Chinese studies reported that patients treated with transarterial chemoembolization with longer leukocyte RTL had a shorter survival time 44, 45. In a Korean cohort containing 49 HCC patients, patients with a higher RTL tumor/non‐tumor ratio had a relatively poorer survival 37, while in a US cohort of 126 HCC patients, there was no association between telomere length and patient survival 38. The discrepancy in these results could be attributed to differences in follow‐up duration, baseline clinicopathologic characteristics, therapeutic schedules, and/or study populations. We noted that in the US cohort, the time interval for selection of these multi‐racial cases was 39 years, and detailed treatment data were lacking or changed over the years 35. It is known that dissimilarity of telomere length is found among multi‐racial populations 11. In this study, as the largest integrative analysis of telomere length in HCC to date, 257 Chinese patients who received curative surgical resection were randomly selected within 1 year, most of whom were HBV‐related cases. In addition, the method used in this study can evaluate cancer cells individually, while others evaluated tumor tissues as a whole. Using internal and external validation datasets, we demonstrated that shortened telomeres in tumor cells were independently and significantly associated with poor clinical outcome in HCC patients.

The tumor microenvironment is increasingly recognized as a significant factor in cancer. In this regard, a recent study showed telomere shortening in cancer‐associated stromal cells 18, 46. Here, we comprehensively investigated non‐tumor cells within their local milieu in HCC, that is, CAF/NTF, TIL/NTIL, and T‐BDEC/P‐BDEC pairs. We found significant telomere variation in CAFs, but not in TILs or T‐BDECs. It has been reported that telomere shortening in fibroblasts can lead to an altered pattern of secreted factors, such as increased production of pro‐inflammatory cytokines and matrix‐degrading proteases 47. Therefore, CAFs with shortened telomeres may boost the progression of HCC 24, relevant to our findings that shorter telomere length in CAFs predicted worse prognosis.

Various methods have been applied to measure telomere lengths. Telomere‐specific FISH facilitated our investigation of telomere lengths in distinct cell types within the tumor microenvironment at single‐cell resolution. Notable advantages of this method have been described previously 26, such as reduced nonspecific binding and more specific telomere information. The direct assessment of telomere lengths in fixed tissue samples should be valuable for validating hypotheses involving telomere shortening in tumorigenesis and progression.

Last but not least, 16 and 22 HCC cases demonstrated markedly heterogeneous telomere lengths in tumor cells and CAFs within individual tumors (termed intratumor cell–cell heterogeneity). This heterogeneity may reflect inconsistent rates of telomere dynamics and variable reactivation of telomerase within subpopulations of cells due to variation in antioxidant protective effects, or differences in telomere preservation/extension mechanisms. Such intratumor heterogeneity was proposed as a major obstacle for curative therapy in HCC 3, 48, inviting new challenges in the molecular understanding of HCC 49.

In conclusion, we revealed significant telomere shortening in HCC cells and CAFs compared with their normal counterparts. More importantly, telomere shortening in cancer cells or CAFs was identified as an independent prognostic indicator for reduced survival and increased recurrence in HCC patients, highlighting the critical role of telomere dysfunction in HCC progression. As such, telomere variation in tumor cells and CAFs within the tumor microenvironment of HCC should be considered as a valuable biomarker in future clinical practice.

## Author contributions statement

LJM and QG conceived and performed most of the experiments. LJM, MD, and ZCW developed the methodology. QG, LZL, AWK, LQD, JYS, XMZ, and YC provided facilities and acquired and managed patients. LJM, QG, and MD analyzed and interpreted data. QG, LJM, XYW, and MD wrote and reviewed the manuscript. LJM, ZBD, LXY, JZ, and JF organized data and constructed databases. QG and XYW supervised the study.

**Abbreviations**
AFP: alpha‐fetoprotein
ALT: alanine transaminase
BCLC: Barcelona Clinic Liver Cancer
CAFs: cancer‐associated fibroblasts
HBsAg: hepatitis B surface antigen
HCC: hepatocellular carcinoma
HCVAb: hepatitis C virus antibody
HR: hazard ratio
IQR: interquartile range
NTF: non‐tumoral fibroblast
OS: overall survival
P‐BDEC: peritumor bile duct epithelial cell
PTIL: peritumor‐infiltrating lymphocyte
T‐BDEC: tumor bile duct epithelial cell
TERTp: telomerase gene promoter
TIL: tumor‐infiltrating lymphocyte
TNM: tumor–node metastasis
TTR: time to recurrence
γ‐GT: γ‐glutamyltransferase

**SUPPLEMENTARY MATERIAL ONLINE**

**Supplementary materials and methods**

**Supplementary figure legends**

**Figure S1.** Measurement of telomere length by ImageJ and identification of cell types in TMA by H&E

**Figure S2.** Representative FISH images of telomere length variation in HCC cells and non‐tumor cells

**Figure S3.** Relative telomere length detected by qPCR

**Figure S4.** Kaplan–Meier curves of OS and TTR according to the median telomere length

**Table S1.** Patient characteristics

**Table S2.** Descriptive statistics of telomere‐specific FISH (*n* = 257)

**Table S3.** Univariate and multivariate analysis of factors associated with OS (*n* = 257)

**Table S4.** Univariate and multivariate analysis of factors associated with TTR (*n* = 257)

## Supporting information


**Supplementary materials and methods**

**Supplementary figure legends**

**Figure S1.** **Measurement of telomere length by ImageJ and identification of cell types in TMA by H&E.** Representative images of the telomere quantitative process, including the original, conversion, normalization, and gradually measurement, are shown. (A) Four images illustrate the intensity of DAPI signals stained for nuclear DNA (top panels) and the intensity of telomere signals in the same field of vision (bottom panels) in HCC cell lines. (B) Images indicate the DAPI signals stained for nuclear DNA (top panels) and matched telomere signals (bottom panels) in HCC tissues. (C) Representative H&E image staining of tumor cells, peritumor liver cells, NTFs, and CAFs (black arrow indicates fibroblasts). (D) Representative H&E images for illustrating PTILs, TILs, P‐BDECs, and T‐BDECs (original magnification ×40). NTFs = non‐tumoral fibroblasts; CAFs = carcinoma‐associated fibroblasts; PTILs = peritumor infiltrate lymphocytes; TILs = tumor infiltrating lymphocytes (arrowhead indicates lymphocyte); P‐BDECs = peritumor bile duct epithelial cells; T‐BDECs = tumor bile duct epithelial cells (black arrow indicates bile duct epithelial cells).

**Figure S2.** **Representative FISH images of telomere length variation in HCC cells and non‐tumor cells. (**A) Tumor cells; (B) cancer‐associated fibroblasts (CAFs); (C) infiltrative lymphocytes; (D) bile duct epithelial cells (BDECs). White asterisks indicate tumor cells; short white arrows indicate CAFs; long white arrows indicate bile duct epithelial cells and white triangles infiltrative lymphocytes. Left panel: DAPI fluorescence; middle panel, Cy3‐PNA telomere probe fluorescence; right panel, merged images of telomere and DAPI (original magnification ×40).

**Figure S3.** **Relative telomere length detected by qPCR. (**A) Shortened RTL was confirmed in tumor compared with adjacent non‐tumor tissues (n = 24). ***p < 0.001. (B) Shortened RTL was validated in CAFs compared with that in NTFs (n = 10). **p < 0.01. CAFs and NTFs were isolated using microbeads as described in the Materials and methods. (C) No significant difference was found in PTILs and TILs (n = 10). PTILs and TILs were isolated using microbeads as described in the Materials and methods. (D) The relative telomere length of tumor cells correlates significantly with the relative TERT mRNA level (n = 64; r = 0.806, p < 0.0001). (E) Representative images showing telomere intensity in paired tumor cells and peritumor liver cells. Case #29: fewer telomere signals in tumor cells than in paired peritumor cells; case #41: stronger telomere signals in tumor cells than in peritumor liver cells. (F) Representative images showing telomere intensity in paired NTFs and CAFs. Case #32: fewer telomere signals in CAFs than in NTFs; case #44: stronger telomere signals in CAFs than in NTFs. Short white arrows indicate NTFs and long white arrows CAFs. Original magnification ×40.

**Figure S4.** **Kaplan–Meier curves of OS and TTR according to the median telomere length. (**A, B) Tumor cells; (C, D) CAFs. Longer telomeres in tumor cells or CAFs were associated with prolonged survival and reduced recurrence. P values were determined by the log‐rank test.

**Table S1.** Patient characteristics

**Table S2.** Descriptive statistics of telomere specific‐FISH (n = 257)

**Table S3.** Univariate and multivariate analysis of factors associated with OS (n = 257)

**Table S4.** Univariate and multivariate analysis of factors associated with TTR (n = 257)
