# Supplementary material for: Telomere length variation in tumor cells and cancer‐associated fibroblasts: potential biomarker for hepatocellular carcinoma
Source: J Pathol. 2017 Oct 13;243(4):407–17. doi: 10.1002/path.4961 (PMC5725724; doi:10.1002/path.4961)
Supplement: Supplementary file 1 — Supplementary materials and methods [file PATH-243-407-s002.docx]

**Supplementary Materials**

**Telomere Length Variation in Tumor cells and Cancer-Associated Fibroblasts: Potential Biomarker for Hepatocellular Carcinoma**

Li-Jie Ma, Xiao-Ying Wang, Meng Duan, Jie-Yi Shi, Liang-Qing Dong, Liu-Xiao Yang, Zhi-Chao Wang, Zhen-Bin Ding, Ai-Wu Ke, Ya Cao, Xiao-Ming Zhang, Jian Zhou, Jia Fan and Qiang Gao

**Supplementary Materials and Methods**

**Cell lines**

Five human HCC cell lines, Huh7 (a well-differentiated and non-metastatic HCC cell line, Japanese Cancer Research Resources Bank), SMMC-7721 (a HCC cell line with low metastatic potential, Chinese Academy of Sciences, Shanghai, China), MHCC-97L, MHCC-97H, and MHCC-LM3 were used in this study. The latter three HCC cell lines were established in our institute, all from the same parental cell line MHCC97, with stepwise pulmonary metastatic potential (MHCC-97L < MHCC-97H < HCC-LM3) as reported elsewhere (23). L-02 (an immortalized human liver cell line) and the cervical cancer cell line Hela were also used. All lines were maintained in Dulbecco’s modified Eagle’s media (DMEM), supplemented with 10% heat-inactivated fetal bovine serum (FBS) and 1% penicillin/streptomycin. All seven cell lines were cultured at 37°C in a humidified incubator in 5% CO_2_.

**Patients and sample collection**

This study was conducted after obtaining informed consent form from patients and ethical approval from Zhongshan Hospital Research Ethics Committee. The median duration of follow-up was 64.5 months (range, 3-74 months). At the end of follow-up, 134 and 149 out of the 257 patients had died and tumor recurrence, respectively. The Edmondson grading system was used to classified degree of tumor differentiation, and the main nodule was selected if the patient had multifocal tumors. The most advanced histologic grade of tumor was used for our study. Overall survival (OS) and time to recurrence (TTR) were defined as the period from the date of the surgery to death and recurrence, respectively. The OS and TTR (in parentheses) rates at 1, 3 and 5 years post-hepatectomy were 80.15% (68.87%), 59.14% (50.19%) and 47.86% (42.02%) for the whole study population.

**Tissue Microarray Construction**

Core samples were selected from representative regions of each tumor by Hematoxylin-eosin (H&E) staining. Duplicate 1-mm diameter cylinders were taken from two distinct areas in tumor center and peritumor noncancerous tissue (a total of four cores) for each case. Sections with 4 um thick were tiled on slides coated with 3-aminopropyltriethoxysilane.

**Cell Lines Blocks Preparation**

Cell lines were cultured to 50 to 75% confluence, and fixed in 10% (v/v) phosphate-buffered formalin at 25°C for 15 hours. Fixed cells were gathered by centrifugation at 500g for 10 minutes, and washed twice in 1 x PBS, and centrifuged again. Then cell precipitates were resuspended in an equal volume of 0.8% agarose buffer for preservation at 42°C. Then, cells were transferred into a 200ul tube and embedded into the paraffin blocks.

**Cell isolation, purification and phenotypic characterization**

Isolation of CD45^+^ leukocytes and ɑ-SMA^+^ fibroblasts from peritumoral liver and HCC tissues using MicroBeads and the MS Column (Miltenyi Biotec, Bergisch Gladbach, Germany) was performed according to the manufacturer’s instructions. Fresh samples of HCC and paired peritumor tissue were obtained from 10 patients undergoing liver resection. Peritumoral and paired HCC tissues were repeatedly rinsed by 1 x phosphate-buffered saline (PBS), trimmed and minced with scalpels in a tissue culture dish, and then enzymatically dissociated in Roswell Park Memorial Institute-1640 (RPMI-1640) medium supplemented with 500 units/mL collagenase D (Invitrogen), and 100 units/mL hyaluronidase (Calbiochem) at 37 °C for 1 hour. The suspension was then filtrated with a diameter of 0.22um disposable sterile filter to eliminate impurities. The suspension was then centrifuged at 500 rpm and resuspended with PBS. Fibroblast antigen–positive cells were isolated from the cell suspension by positive selection using anti-fibroblast MicroBeads and the MS Column (Miltenyi Biotec) according to the manufacturer’s instructions. Isolated cells were resuspended in PBS and placed in 1.5 ml tube. CAFs and non-tumoral fibroblasts (NTFs) isolated from human HCC tumor and paired non-tumor tissues were immunophenotypically characterized by flow cytometry for a-SMA, CD34 and CD45. Purity of the isolated fibroblast population was > 99% (data not show). For leukocytes, positive cells were isolated from the cell suspension by positive selection using anti-human CD45 MicroBeads and the MS Column (Miltenyi Biotec) according to the manufacturer’s instructions. Infiltrative leukocytes isolated from tumor and nontumor tissues were also immunophenotypically characterized by flow cytometry for CD45. Purity of the isolated leukocytes population was > 99% (data not show).

**Flow cytometric analysis**

Fluorescein isothiocyanate (FITC)-labeled a-SMA and allophy-cocyanin (APC)-labeled CD45 antibodies were purchased from R&D. Cells were permeabilized and incubated with specific antibodies after sorting. Flow cytometry was performed on a FACS Aria II (BD Immunocytometry Systems) according to the manufacturer’s instructions, and analyzed with FlowJo software version 7.6.1(Tree Star).

**Measurement of relative telomere length by real-time quantitative PCR**

“Genomic DNA was extracted from paired peritumoral liver and HCC tissues from 24 patients, as well as isolated leukocytes and fibroblasts from 10 patients using DNeasy Blood & Tissue Kit (Qiagen, Chatsworth, California, USA) according to the manufacturer’s protocols. The mRNA was extracted from paired peritumoral liver and HCC tissues from another 64 patients.” As previously described, two pairs of primers were used for measuring the copy numbers of telomere and single copy gene 36B4. The sequence of primers for qPCR was as follows: Telomere forward primer: 5’-ACACTAAGGTTTGGGTTT-GGGTTTGGGTTTGGGTTAGTGT-3’, Telomere reverse primer: 5’-TGTTAGGTATCCCTATCCCTATCCC- TATCCCTATCCCTAACA-3’. TERT forward primer: CTCCAGGCACAACGAACGC. TERT reverse primer: GATCTCCTCACGCAGACGGT. 36B4 forward primer: 5’-CAGCAAGTGGGAAGGTGTAATCC-3’, 36B4 reverse primer: 5’-CCCATTCTATCATCAACGGGTACAA-3’. Briefly, PCR (for 20ul system) comprised for gene-specic amplication consisted of mix containing 1 × SYBR green master mix (TaKaRa), 10 nM forward primer, 10 nM reverse primer, and 4 ng of genomic DNA. Thermal cycling for PCR is 95 °C for 30 s, followed by 35 cycles of 94 °C for 30 s, 56 °C (for telomere amplication) for 30 s or 58 °C for 1 minute, and 72 °C for 50 s with signal acquisition. The copy numbers of telomere and 36B4 gene were run in separate 384 well plates on Applied Biosystems 7900HT Sequence Detection System (Foster City, California, USA), and triplicate reactions were performed. The ratio of telomere/single copy gene copy numbers (T/S) is proportional to the average telomere length per cell as previously described. The calibrated T/S ratio was then used as the measurement of relative telomere lengths (RTLs).

**Telomere quantitative fluorescent in situ hybridization**

Briefly, sections were deparaffinized and hydrated successively, then washed by deionized water and followed by deionized water with 0.1% Tween-20 (catalog No. SC-29113; Santa Cruz). 14 minutes of heat-induced antigen retrieval was done in a steamer with citrate buffer (catalog No. XY-0016; eBioscience). Then slides were infiltrated in PBS with Tween (PBST; catalog No. 9809S; CST), and rinsed, dehydrated, air-dried successively. 30ul of hybridization reaction (catalog No. 11717472001; Roche) mixture, which comprised Cy3-labeled telomere-specific peptide nucleic acid (PNA) hybridization probe (catalog No. F1002; Panagene, Korea), was coverslipped by incubation and denaturation at 83^o^C for 4 minutes. Slides were then hybridized for 2 hours at normal temperature away from light. After that, slides were washed twice within PNA wash buffer. Then slides were infiltrated, rinsed, desiccated and counterstained with 4’-6-diamidino-2-phenylindole (DAPI) (catalog No. D009501; Sigma). In the end, TMA sections were mounted with fluorescent mounting media (catalog No. E675009; BBI) for followed imaged.

**Microscopy Assessment**

Fluorescence excitation/emission filters applied as follows: Cy3 excitation, 546 nm/10 nm BP; emission, 578 nm LP (Olympus Inc.); DAPI excitation, 330 nm; emission, 400 nm via a fluorescence set (Olympus Inc.). Fluorescent images were captured with a cooled charge-coupled device camera (OLYMPUS DP73, Japan). Typically, integration times ranged from 400 milliseconds to 800 milliseconds for Cy3 (telomere) signal capture and 50 milliseconds to 100 milliseconds for DAPI counter-stain. Before the formal imaging, optimum exposure times were designated to hold the remaining exposure times constant thereafter, which enabled all cells were exposed within a comparison set experienced identical exposure time and avoided the saturation of fluorescence signal. In all exposures, telomeric intensities were within the linear response range of the charge-coupled device camera, which was validated by use of fluorescent microbead intensity standards (InSpeck microscope image intensity calibration fluorescent microspheres; Molecular Probes). For the two color channels, separate grayscale images were captured simultaneously.

**Analysis of Telomere Length**

Briefly, images were first converted to type of 8-bit and inverted before the background subtraction, sharpening, and enhancement. Then through calibrating, scale setting and measurements setting, matched nuclear DNA and telomeric grayscale TIFF image files were normalized. Measurement field value was selected by the option of threshold. Data were gathered on an individual cell basis. Thirty cells in each interest region was casually selected and analyzed for each cell type. Less than thirty cells were involved as in previously studies. DAPI signal or telomeric signal was finally measured by the option of analyze and measurement. The fluorescence intensity was defined as the ratio of total optical density to total fluorescence area in the image, and the median ratio of telomere signal sum to DAPI signal sum was defined as telomere length. In all representative images, DAPI (blue) was used to identify nuclei and Cy3-labeled telomere-specific peptide nucleic acid probe (red) was binded the telomere DNA. Noticeably, the centromere DNA, stained with the Cy3-labeled centromere-specific peptide nucleic acid probe as the control, was removed from the image to highlight the variations in the telomere lengths. Telomere length was analyzed in six cell types: cancer cells, adjacent liver cells, CAFs, NTFs, infiltrating lymphocytes and bile duct epithelial cells (BDECs). Identification of eight cell types was characterized by the H&E staining in serial slides signed by the pathologist. A few cases of spots did not comprise cancer cells or adjacent liver cells as a result of the exhaustion during serial sectioning. In these TMA cores, we were capable of evaluating the remaining cell types.

**Statistical Analyses**

The analysis of the association between variables was conducted using the Mann Whitney U test, Student t test, Chi-square test, or one-way ANOVA test when appropriate. Univariate analysis was based on the Kaplan-Meier method using the log-rank test. Cox proportional hazards regression was used for multivariate analyses. SPSS software (IBM, Armonk, NY, USA) and Graph Pad Prism 6.0 (Graph Pad software Inc., San Diego, CA, USA) were applied for data analyses.
