# Supplementary material for: Telomere length variation in tumor cells and cancer‐associated fibroblasts: potential biomarker for hepatocellular carcinoma
Source: J Pathol. 2017 Oct 13;243(4):407–17. doi: 10.1002/path.4961 (PMC5725724; doi:10.1002/path.4961)
Supplement: Supplementary file 2 — Supplementary figure legends [file PATH-243-407-s008.docx]

**Supplementary Figure legends**

**Figure S1.**

**Measurement of Telomere Length by Image J and identification of cell types in TMA by H&E.** Representative images of telomere quantitative process, including the original, conversion, normalization, and gradually measurement, are shown. A, Four images illustrate the intensity of DAPI signals stained for nuclear DNA (Top panels) and intensity of telomere signals in the same field of vision (Bottom panels) in HCC cell lines. B, Images indicate the DAPI signals stained for nuclear DNA (Top panels) and matched telomere signals (Bottom panels) in HCC tissues. C, Representative H&E images staining of tumor cells, peritumor liver cells, NTFs and CAFs (black arrow means fibroblasts). D, Representative H&E images for illustrating PTILs, TILs, P-BDECs and T-BDECs. (Magnification × 40). Abbreviation: NTFs: non-tumoral fibroblasts; CAFs: carcinoma-associated fibroblasts; PTILs: peritumor infiltrate lymphocytes; TILs: tumor infiltrate lymphocytes (triangular arrow means lymphocyte); P-BDECs: peritumor bile duct epithelial cells; T-BDECs: tumor bile duct epithelial cells (black arrow means bile duct epithelial cells).

**Figure S2.**

**Representative FISH images of telomere length variation in HCC cells and non-tumor cells.** A, Tumor cells. B, Cancer-associated fibroblasts (CAFs). C, Infiltrative lymphocytes. D, Bile duct epithelial cells (BDECs). White asterisks indicate tumor cells, short white arrows indicate CAFs, long white arrows highlight bile duct epithelial cells and white triangles represent infiltrative lymphocytes. Left panel, DAPI fluorescence. Middle panel, Cy3-PNA telomere probe fluorescence. Right panel, merged images of telomere and DAPI. (Magnification × 40).

**Figure S3.**

**Relative telomere length detected by qPCR.** A, Shortened RTL was confirmed in tumor compared with adjacent non-tumor tissues (n=24). *P* < 0.001. B, Shortened RTL was validated in CAFs compared with that in NTFs (n=10). *P* < 0.01. CAFs and NTFs were isolated using microbeads as described in the Supplementary materials and methods. C, No significant difference was found in PTILs and TILs (n=10). PTILs and TILs were isolated using microbeads as described in the Supplementary materials and methods. D, The relative telomere length of tumor cells significantly correlates with the relative *TERT* mRNA level (n = 64; r = 0.806, *P* < 0.0001). E, Representative images showing telomere intensity in paired tumor cells and peritumor liver cells. Case #29: fewer telomere signals in tumor cells than paired peritumor cells; Case #41: stronger telomere signals in tumor cells than peritumor liver cells. F, Representative images showing telomere intensity in paired NTFs and CAFs. Case #32: fewer telomere signals in CAFs than NTFs; Case #44: stronger telomere signals in CAFs than NTFs. Short white arrows indicate NTFs and long white arrows highlight CAFs. (Magnification × 40).

**Figure S4.**

**Kaplan–Meier curves of OS and TTR according to the median telomere length.** A and B, tumor cells. C and D, CAFs. Longer telomere in tumor cells or CAFs were associated with prolonged survival and reduced recurrence. *P* values were determined by the log-rank test.
