# Supplementary figures and images for: Telomere length variation in tumor cells and cancer‐associated fibroblasts: potential biomarker for hepatocellular carcinoma
Source: J Pathol. 2017 Oct 13;243(4):407–17. doi: 10.1002/path.4961 (PMC5725724; doi:10.1002/path.4961)

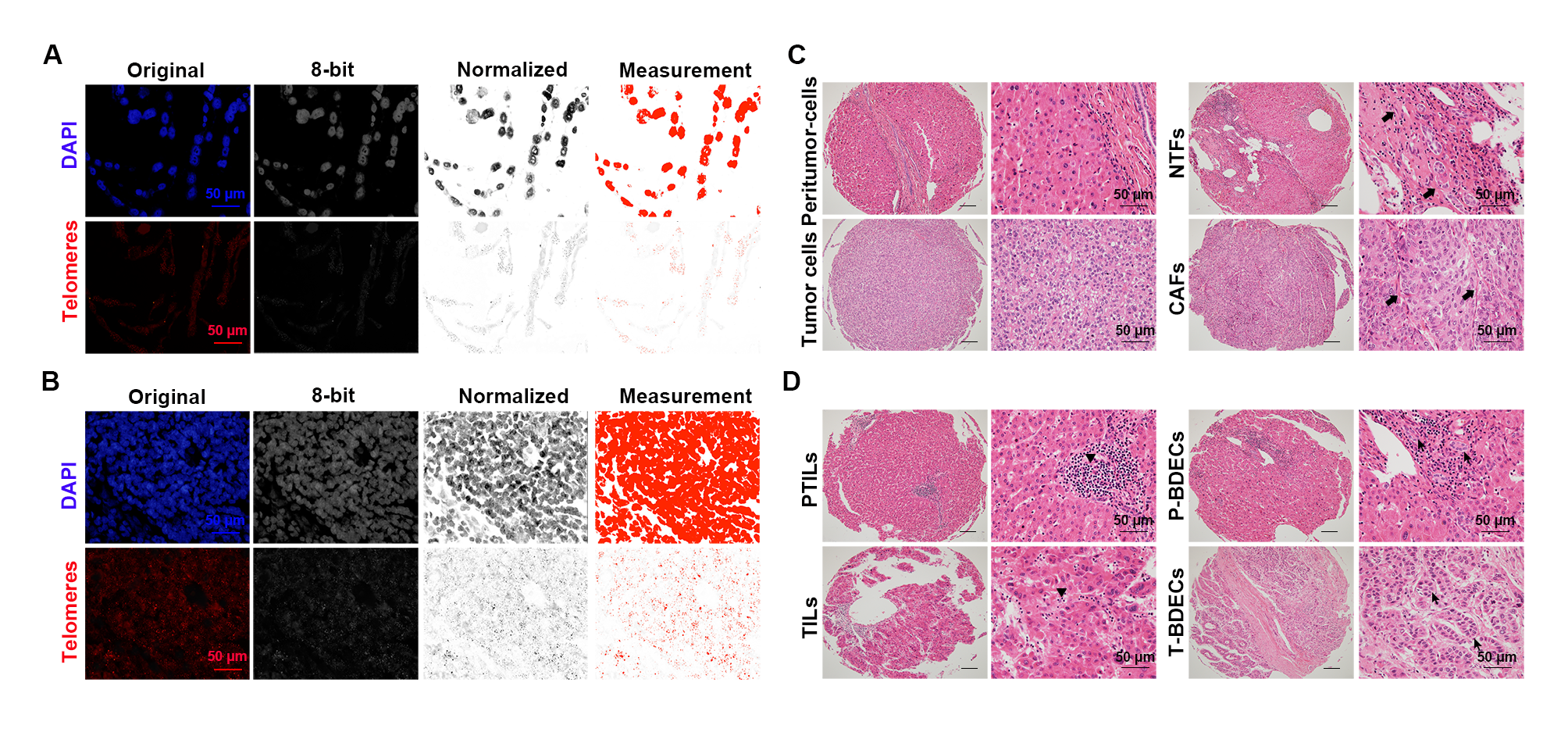

Supplement: Supplementary file 3 — Figure S1. Measurement of telomere length by ImageJ and identification of cell types in TMA by H&E. Representative images of the telomere quantitative process, including the original, conversion, normalization, and gradually measurement, are shown. (A) Four images illustrate the intensity of DAPI signals stained for nuclear DNA (top panels) and the intensity of telomere signals in the same field of vision (bottom panels) in HCC cell lines. (B) Images indicate the DAPI signals stained for nuclear DNA (top panels) and matched telomere signals (bottom panels) in HCC tissues. (C) Representative H&E image staining of tumor cells, peritumor liver cells, NTFs, and CAFs (black arrow indicates fibroblasts). (D) Representative H&E images for illustrating PTILs, TILs, P‐BDECs, and T‐BDECs (original magnification ×40). NTFs = non‐tumoral fibroblasts; CAFs = carcinoma‐associated fibroblasts; PTILs = peritumor infiltrate lymphocytes; TILs = tumor infiltrating lymphocytes (arrowhead indicates lymphocyte); P‐BDECs = peritumor bile duct epithelial cells; T‐BDECs = tumor bile duct epithelial cells (black arrow indicates bile duct epithelial cells). [file PATH-243-407-s010.tif]

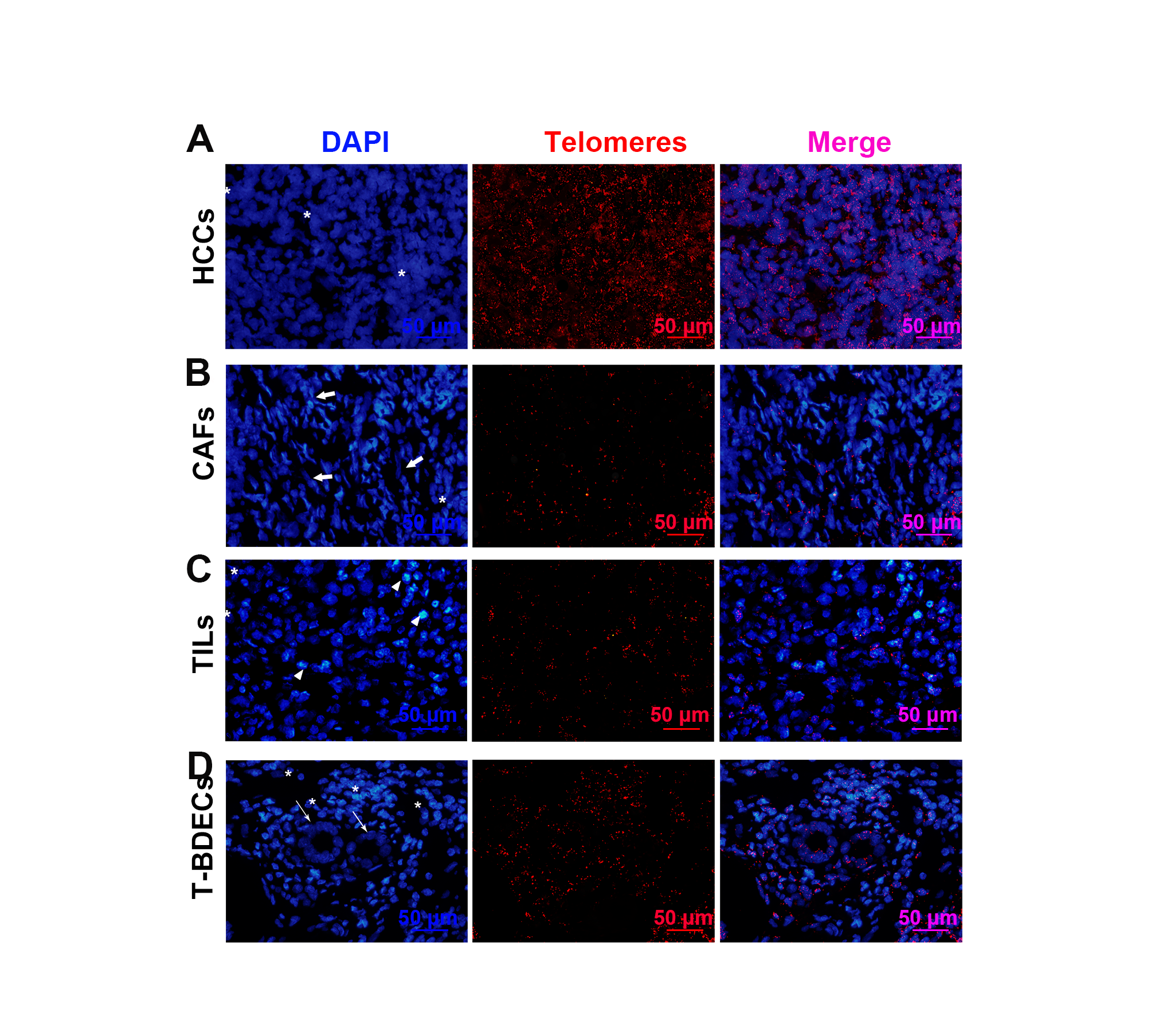

Supplement: Supplementary file 4 — Figure S2. Representative FISH images of telomere length variation in HCC cells and non‐tumor cells. (A) Tumor cells; (B) cancer‐associated fibroblasts (CAFs); (C) infiltrative lymphocytes; (D) bile duct epithelial cells (BDECs). White asterisks indicate tumor cells; short white arrows indicate CAFs; long white arrows indicate bile duct epithelial cells and white triangles infiltrative lymphocytes. Left panel: DAPI fluorescence; middle panel, Cy3‐PNA telomere probe fluorescence; right panel, merged images of telomere and DAPI (original magnification ×40). [file PATH-243-407-s001.tif]

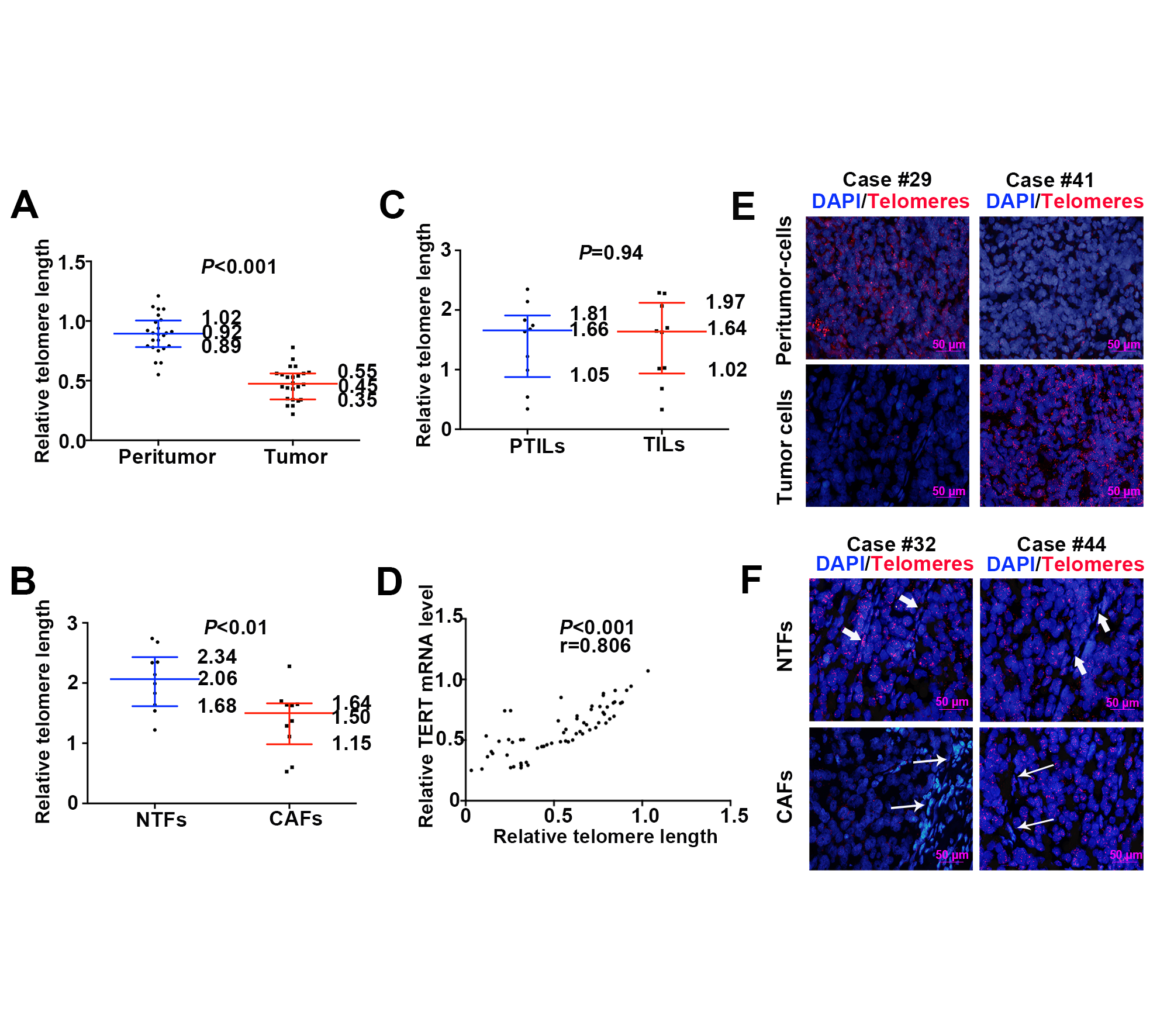

Supplement: Supplementary file 5 — Figure S3. Relative telomere length detected by qPCR. (A) Shortened RTL was confirmed in tumor compared with adjacent non‐tumor tissues (n = 24). ***p < 0.001. (B) Shortened RTL was validated in CAFs compared with that in NTFs (n = 10). **p < 0.01. CAFs and NTFs were isolated using microbeads as described in the Materials and methods. (C) No significant difference was found in PTILs and TILs (n = 10). PTILs and TILs were isolated using microbeads as described in the Materials and methods. (D) The relative telomere length of tumor cells correlates significantly with the relative TERT mRNA level (n = 64; r = 0.806, p < 0.0001). (E) Representative images showing telomere intensity in paired tumor cells and peritumor liver cells. Case #29: fewer telomere signals in tumor cells than in paired peritumor cells; case #41: stronger telomere signals in tumor cells than in peritumor liver cells. (F) Representative images showing telomere intensity in paired NTFs and CAFs. Case #32: fewer telomere signals in CAFs than in NTFs; case #44: stronger telomere signals in CAFs than in NTFs. Short white arrows indicate NTFs and long white arrows CAFs. Original magnification ×40. [file PATH-243-407-s004.tif]
