# Supplementary material for: Telomere length variation in tumor cells and cancer‐associated fibroblasts: potential biomarker for hepatocellular carcinoma
Source: J Pathol. 2017 Oct 13;243(4):407–17. doi: 10.1002/path.4961 (PMC5725724; doi:10.1002/path.4961)
Supplement: Supplementary file 7 — Table S1. Patient characteristics [file PATH-243-407-s005.docx]

**Table. S1.** Patient characteristics.

| **Characteristics** | **Results** |
| --- | --- |
| Age (years) (Median,Range) | 49.3(10-79) |
| Sex (Male/Female) | 219/38 |
| Underlying disease (HBV/HCV/HBV+HCV/No) | 248/9/3/2 |
| ɑ-Fetoprotein (ng/ml) (Median,Range) | 6671.8(0-60500.0) |
| Alanine aminotransferase (U/L) (Median,Range) | 36(10-561) |
| γ-glutamyltranspeptidase (U/L) (Median,Range) | 63(13-648) |
| Total bilirubin (μmol/L) (Median,Range) | 13.3(4.4-37.1) |
| Liver cirrhosis (Yes/No) | 232/25 |
| Maximum diameter (cm) (≤5/>5) | 136/121 |
| Tumor encapsulation (Complete/None) | 143/114 |
| Tumor number (1/2-3/>3) | 212/43/2 |
| Tumor differentiation (I-II/III-IV) | 181/76 |
| Vascular invasion number (<1/1-3/>3) | 167/68/22 |
| Hepatic hilar lymph node metastasis (Yes/No) | 5/252 |
| UICC TNM stage (I/II/III) | 145/61/51 |
| BCLC stage (0/A/B/C) | 21/80/65/91 |
| Site of recurrence (Yes/No) | 120/137 |

**Abbreviation:** HBV, Hepatitis B Virus; HCV, Hepatitis C Virus; UICC, International Union Against Cancer; BCLC: Barcelona Clinic Liver Cancer.
