# Supplementary material for: Telomere length variation in tumor cells and cancer‐associated fibroblasts: potential biomarker for hepatocellular carcinoma
Source: J Pathol. 2017 Oct 13;243(4):407–17. doi: 10.1002/path.4961 (PMC5725724; doi:10.1002/path.4961)
Supplement: Supplementary file 8 — Table S2. Descriptive statistics of telomere specific‐FISH (n = 257) [file PATH-243-407-s009.docx]

**Table S2.** Descriptive Statistics of telomere specific-FISH (*n* = 257).

| **Variables*** | **Mean** | **SE** | **Median** | **IQR** |
| --- | --- | --- | --- | --- |
| **Intratumor** |  |  |  |  |
| Tumor cells | 14.50 | 0.77 | 11.74 | 3.32-22.16 |
| CAFs | 47.06 | 1.64 | 42.82 | 26.81-61.10 |
| TILs | 24.09 | 1.27 | 19.52 | 5.53-36.83 |
| T-BDECs | 16.54 | 0.87 | 13.32 | 3.78-25.15 |
| **Peritumor** |  |  |  |  |
| Peritumor cells | 28.98 | 1.16 | 25.90 | 17.78-35.43 |
| NTFs | 63.83 | 2.15 | 63.31 | 34.45-87.21 |
| PTILs | 22.98 | 1.21 | 18.61 | 5.27-35.12 |
| P-BDECs | 15.69 | 0.82 | 12.71 | 3.60-23.99 |

**Abbreviation:** CAFs: cancer-associated fibroblasts; NTFs: non-tumoral fibroblasts；IQR: interquartile range; TILs: tumor-infiltrating lymphocytes; PTILs: peritumor infiltrate lymphocytes; T-BDECs: tumor bile duct epithelial cells; P-BDECs: peritumor bile duct epithelial cells.
