# Supplementary material for: Telomere length variation in tumor cells and cancer‐associated fibroblasts: potential biomarker for hepatocellular carcinoma
Source: J Pathol. 2017 Oct 13;243(4):407–17. doi: 10.1002/path.4961 (PMC5725724; doi:10.1002/path.4961)
Supplement: Supplementary file 9 — Table S3. Univariate and multivariate analysis of factors associated with OS (n = 257) [file PATH-243-407-s006.docx]

**Table S3.** Univariate and multivariate analysis of factors associated with OS (*n* = 257).

|  |  | **A** | | | **B** | | | **C** | | |
| --- | --- | --- | --- | --- | --- | --- | --- | --- | --- | --- |
|  | **Univariate** | **Multivariate** | | | **Multivariate** | | | **Multivariate** | | |
| **Variables** | ***P*** | **HR** | **95%CI** | ***P*** | **HR** | **95%CI** | ***P*** | **HR** | **95%CI** | ***P*** |
| Age, years (>51vs.≤51) | 0.887 |  |  | NA |  |  | NA |  |  | NA |
| Gender (male vs. female) | 0.508 |  |  | NA |  |  | NA |  |  | NA |
| HBsAg (positive vs. negative) | 0.160 |  |  | NA |  |  | NA |  |  | NA |
| HCVAb (positive vs. negative) | 0.287 |  |  | NA |  |  | NA |  |  | NA |
| Serum AFP, ng/ml (>20vs.≤20) | **0.001** | **1.700** | **1.014-2.851** | **0.044** | **1.726** | **1.027-2.900** | **0.039** | **1.831** | **1.086-3.085** | **0.023** |
| Serum ALT, U/L (>75vs.≤75) | 0.316 |  |  | NA |  |  | NA |  |  | NA |
| Serum γ-GT, U/L (>54vs.≤54) | **0.017** |  |  | NS |  |  | NS |  |  | NS |
| Liver cirrhosis (yes vs. no) | 0.454 |  |  | NA |  |  | NA |  |  | NA |
| Tumor size (cm) (>5 vs. ≤5) | **<0.001** |  |  | NS |  |  | NS |  |  | NS |
| Tumor multiplicity (multiple vs. single) | 0.212 |  |  | NA |  |  | NA |  |  | NA |
| Tumor differentiation (poor vs. well) | **<0.001** | **1.711** | **1.162-2.519** | **0.007** | **1.551** | **1.053-2.286** | **0.026** | **1.641** | **1.114-2.418** | **0.012** |
| Vascular invasion (yes vs. no) | **<0.001** |  |  | NS |  |  | NS |  |  | NS |
| TNM stage (III-II vs. I) | **<0.001** |  |  | NS |  |  | NS |  |  | NS |
| BCLC stage (B-C vs. 0-A) | **<0.001** | **3.154** | **1.598-6.225** | **0.001** | **3.283** | **1.676-6.431** | **0.001** | **3.490** | **1.765-6.902** | **<0.001** |
| Tumor cells (Shorter vs. Longer) | **<0.001** | **2.555** | **1.616-4.039** | **<0.001** |  |  |  |  |  |  |
| CAFs (Shorter vs. Longer) | **<0.001** |  |  |  | **2.219** | **1.444-3.411** | **<0.001** |  |  |  |
| Combination of tumor cells and CAFs^#^ |  | | | | | | | | | |
| Overall | **<0.001** |  |  |  |  |  |  | NA | NA | **<0.001** |
| II vs. I | 0.088 |  |  |  |  |  |  | 1.268 | 0.496-3.237 | 0.620 |
| III vs. I | **0.041** |  |  |  |  |  |  | 1.047 | 0.460-2.380 | 0.913 |
| IV vs. I | **<0.001** |  |  |  |  |  |  | **2.633** | **1.566-4.427** | **<0.001** |

**NOTE: ^#^** Patients were divided into four groups based on their telomeres densities of tumor cells and CAFs: Group I, longer telomere in tumor cells and longer telomere in CAFs; Group II, longer telomeres in tumor cells and shorter telomeres in CAFs; Group III, shorter telomeres in tumor cells and longer telomeres in CAFs; Group IV, shorter telomeres in tumor cells and shorter telomeres in CAFs. Group I was considered as the control.

Kaplan–Meier method (log-rank test) in SPSS was performed to accomplish univariate analysis. Multivariate analysis was evaluated using the Cox multivariate proportional hazard regression model with stepwise analysis.

* Numbers in bold indicate that the *P* value is significant.

**Abbreviations:** OS, overall survival; CAFs, cancer-associated fibroblasts; HBsAg, hepatitis B surface antigen; HCVAb, hepatitis C virus antibody; AFP, alpha-fetoprotein; ALT, alanine transaminase; γ-GT, γ-glutamyltransferase; TNM, tumor-node-metastasis; BCLC, Barcelona Clinic Liver Cancer; HR, hazard ratio; CI, confidential interval; NA, not applicable; NS, not significant.
