# Supplementary material for: Telomere length variation in tumor cells and cancer‐associated fibroblasts: potential biomarker for hepatocellular carcinoma
Source: J Pathol. 2017 Oct 13;243(4):407–17. doi: 10.1002/path.4961 (PMC5725724; doi:10.1002/path.4961)
Supplement: Supplementary file 10 — Table S4. Univariate and multivariate analysis of factors associated with TTR (n = 257) [file PATH-243-407-s007.docx]

**Table S4.** Univariate and multivariate analysis of factors associated with TTR (*n* = 257).

|  |  | **A** | | | **B** | | | **C** | | |
| --- | --- | --- | --- | --- | --- | --- | --- | --- | --- | --- |
|  | **Univariate** | **Multivariate** | | | **Multivariate** | | | **Multivariate** | | |
| **Variables** | ***P*** | **HR** | **95%CI** | ***P*** | **HR** | **95%CI** | ***P*** | **HR** | **95%CI** | ***P*** |
| Age, years (>51vs.≤51) | 0.580 |  |  | NA |  |  | NA |  |  | NA |
| Gender (male vs. female) | 0.819 |  |  | NA |  |  | NA |  |  | NA |
| HBsAg (positive vs. negative) | 0.129 |  |  | NA |  |  | NA |  |  | NA |
| HCVAb (positive vs. negative) | 0.204 |  |  | NA |  |  | NA |  |  | NA |
| Serum AFP, ng/ml (>20vs.≤20) | **0.015** |  |  | NS |  |  | NS |  |  | NS |
| Serum ALT, U/L (>75vs.≤75) | 0.256 |  |  | NA |  |  | NA |  |  | NA |
| Serum γ-GT, U/L (>54vs.≤54) | 0.061 |  |  | NS |  |  | NS |  |  | NS |
| Liver cirrhosis (yes vs. no) | 0.878 |  |  | NA |  |  | NA |  |  | NA |
| Tumor size (cm) (>5 vs. ≤5) | **<0.001** |  |  | NS |  |  | NS |  |  | NS |
| Tumor multiplicity (multiple vs. single) | 0.065 |  |  | NA |  |  | NA |  |  | NA |
| Tumor differentiation (poor vs. well) | **0.010** |  |  | NS |  |  | NS |  |  | NS |
| Vascular invasion (yes vs. no) | **<0.001** |  |  | NS |  |  | NS |  |  | NS |
| TNM stage (III-II vs. I) | **<0.001** | **1.664** | **1.051-2.634** | **0.030** | **1.613** | **1.017-2.561** | **0.042** | **1.664** | **1.048-2.642** | **0.031** |
| BCLC stage (B-C vs. 0-A) | **<0.001** | **2.438** | **1.331-4.467** | **0.004** | **2.499** | **1.371-4.555** | **0.003** | **2.640** | **1.440-4.841** | **0.002** |
| Tumor cells (Shorter vs. Longer) | **0.002** | **1.755** | **1.168-2.637** | **0.007** |  |  |  |  |  |  |
| CAFs (Shorter vs. Longer) | **0.010** |  |  |  | **1.610** | **1.086-2.388** | **0.018** |  |  |  |
| Combination of tumor cells and CAFs^#^ |  | | | | | | | | | |
| Overall | **0.024** |  |  |  |  |  |  | NA | NA | **0.022** |
| II vs. I | 0.317 |  |  |  |  |  |  | 0.990 | 0.400-2.447 | 0.982 |
| III vs. I | 0.359 |  |  |  |  |  |  | 0.764 | 0.338-1.731 | 0.519 |
| IV vs. I | **0.002** |  |  |  |  |  |  | **1.715** | **1.097-2.682** | **0.018** |

**NOTE: ^#^** Patients were divided into four groups based on their telomeres densities of tumor cells and CAFs: Group I, longer telomere in tumor cells and longer telomere in CAFs; Group II, longer telomeres in tumor cells and shorter telomeres in CAFs; Group III, shorter telomeres in tumor cells and longer telomeres in CAFs; Group IV, shorter telomeres in tumor cells and shorter telomeres in CAFs. Group I was considered as the control.

Kaplan–Meier method (log-rank test) in SPSS was performed to accomplish univariate analysis. Multivariate analysis was evaluated using the Cox multivariate proportional hazard regression model with stepwise analysis.

* Numbers in bold indicate that the *P* value is significant.

**Abbreviations:** TTR, time to recurrence; CAFs, cancer-associated fibroblasts; HBsAg, hepatitis B surface antigen; HCVAb, hepatitis C virus antibody; AFP, alpha-fetoprotein; ALT, alanine transaminase; γ-GT, γ-glutamyltransferase; TNM, tumor-node-metastasis; BCLC, Barcelona Clinic Liver Cancer; HR, hazard ratio; CI, confidential interval; NA, not applicable; NS, not significant.
